# INCREASED LIFESPAN THROUGH ALTERED GCN4 / ATF-5 IN S. CEREVISIAE AND C. ELEGANS

**DOI:** 10.1093/geroni/igz038.2295

**Published:** 2019-11-08

**Authors:** Mark McCormick, Christine Robbins, Olivia Heath, Marissa Westenskow

**Affiliations:** University of New Mexico Health Sciences Center, Albuquerque, New Mexico, United States

## Abstract

In a whole-genome screen for deletions that increase lifespan in S. cerevisiae, we identified increased Gcn4 signaling as a mediator of increased lifespan. Gcn4 is a nutrient-responsive transcription factor whose entire pathway is functionally conserved from yeast through humans. Accumulation of uncharged tRNAs has been shown to upregulate Gcn4, and its mammalian ortholog, ATF4. Here we demonstrate that chemical inhibitors of tRNA synthetases significantly extend lifespan in both yeast and the nematode C. elegans, in a dose- and Gcn4-dependent manner.

